# Molecular perspectives in hypertrophic heart disease: An epigenetic approach from chromatin modification

**DOI:** 10.3389/fcell.2022.1070338

**Published:** 2022-11-29

**Authors:** Fernando Lizcano, Lizeth Bustamante

**Affiliations:** ^1^ Center of Biomedical Investigation, Universidad de La Sabana (CIBUS), Campus Puente del Comun, Autopista Norte de Bogota, Chia, Colombia; ^2^ Fundación Cardio-Infantil IC, Bogotá, Colombia; ^3^ Universidad del Rosario School of Medicine and Health Sciences, Bogotá, Colombia

**Keywords:** histone covalent modifications, DNA methylation, non-coding RNA, chromatin remodelers, cardiac hypertrophy (CH)

## Abstract

Epigenetic changes induced by environmental factors are increasingly relevant in cardiovascular diseases. The most frequent molecular component in cardiac hypertrophy is the reactivation of fetal genes caused by various pathologies, including obesity, arterial hypertension, aortic valve stenosis, and congenital causes. Despite the multiple investigations performed to achieve information about the molecular components of this pathology, its influence on therapeutic strategies is relatively scarce. Recently, new information has been taken about the proteins that modify the expression of fetal genes reactivated in cardiac hypertrophy. These proteins modify the DNA covalently and induce changes in the structure of chromatin. The relationship between histones and DNA has a recognized control in the expression of genes conditioned by the environment and induces epigenetic variations. The epigenetic modifications that regulate pathological cardiac hypertrophy are performed through changes in genomic stability, chromatin architecture, and gene expression. Histone 3 trimethylation at lysine 4, 9, or 27 (H3-K4; -K9; -K27me3) and histone demethylation at lysine 9 and 79 (H3-K9; -K79) are mediators of reprogramming in pathologic hypertrophy. Within the chromatin architecture modifiers, histone demethylases are a group of proteins that have been shown to play an essential role in cardiac cell differentiation and may also be components in the development of cardiac hypertrophy. In the present work, we review the current knowledge about the influence of epigenetic modifications in the expression of genes involved in cardiac hypertrophy and its possible therapeutic approach.

## Introduction

During embryonal development, the heart is one of the first organs to acquire an independent function. The genes that control the growth and differentiation of cardiac cells are influenced by multiple factors that modify normal development (Reeves and Whellan, 2010). In eukaryotes, DNA is bound to nuclear proteins forming chromatin. The functional structure of chromatin is the nucleosome, composed of a histone octamer (H2a, H2b, H3, H4) and about 147 base pairs of DNA. The nucleosome has the function of regulating gene expression, conditioned by the intensity of histone binding to DNA (Lai and Pugh, 2017). In the last years, many studies have demonstrated the abilities of proteins with enzymatic faculty to modify the structure of the nucleosome and give way to processes such as gene expression (Liu and Tang, 2019). All the studies of heritable changes in gene expression without modification in DNA sequences comprise epigenetics. The epigenetics modifications are defined as the variation of DNA methylation in residues of cytosine closed to the promoter areas, the covalent modification of histones, the variation of chromatin induced by the remodeling proteins, and the non-coding RNA (Lei et al., 2021). The enzymatic modifications are reversible and are conditioned by intra and extracellular factors that control gene expression.

**FIGURE 1:**
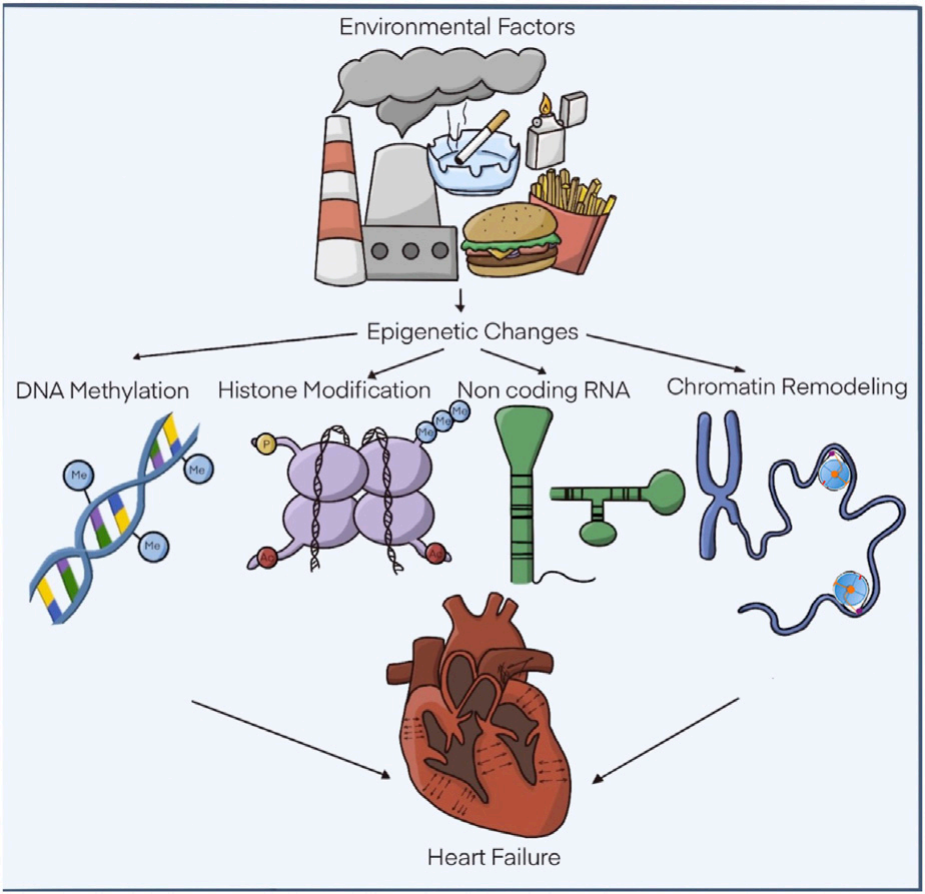
Epigenetic modifications related to hypertrophic cardiomyopathy. The functional alterations of the heart are subject to a series of external factors that influence the recognized epigenetic modifications. DNA methylation, covalent modifications of histones. non-coding RNAs and chromatin remodelers. Modified: Liu, C.-F. et al. J Am Coll Cardiol Basic Trans Science. 2019; 4 (8):976-93.

Even some configurations, such as the trimethylation of histone 3 in lysine 9 residues (H3K9me3), which was considered an irreversible modification, characteristic of heterochromatin, can be modified by external factors (Trojer and Reinberg, 2007). The epigenetic modification that causes cardiac failure is a relatively new field of research (Millan-Zambrano et al., 2022). In the case of cardiac hypertrophy, the response of cardiac cells expands due to an internal or external affectation that has reduced the functional capacity (Figure 1). Heart failure (HF) occurs when the myocardium undergoes structural and functional remodeling and is the primary basis for mortality and the leading cause of death worldwide (Movassagh et al., 2011). HF is the common end point of various cardiovascular conditions, such as cardiac hypertrophy, myocardial infarction, and myocardial ischemia. Hence, understanding how epigenetic regulations are involved in HF may open a new perspective for translational research into new diagnostic tools, novel drug design, and discovery strategies (Nakamura and Sadoshima, 2020).

In human and animal models, pathologically hypertrophied ventricular cardiomyocytes reactivate genes usually expressed in high concentrations during fetal life. This “fetal genetic program” includes genes such as ANP (Atrial Natriuretic peptide), BNP (Brain Natriuretic peptide), and fetal isoforms of myosin heavy chain (MHC) (Mohamed et al., 2016). Additionally, fetal metabolic behavior is resumed, decreasing the rate of fatty acid oxidation, and increasing the rate of glucose oxidation (LaPointe, 2005). Other physiological changes at the cellular level include sarcomere reorganization, alterations in calcium homeostasis, and changes in contractility and relaxation associated with cardiomyocyte death with subsequent fibrosis and electrical remodeling (Meder et al., 2017). Given the broadest number of molecules described with the faculty to modify the structure of chromatin. Factors with the capacity to alter the DNA and chromatin can be grouped as “readers,” “writers,” and “erasers” by how proteins interact with DNA and histones.

The contribution of epigenetic changes to cardiovascular disease is less well understood than other pathologies such as cancer. However, some recent observations on the influence of histone-modifying proteins have motivated particular interest. The possibility exists that focal or chronic hypoxic conditions in cardiovascular diseases may affect JmjC/KDM (Lysine Demethylases) levels and activity and, thus, histone methylation status. Here we discuss evidence demonstrating associations of different epigenetic circumstances that can increase cardiovascular disease progression.

## DNA methylation

Certain regions of the genome contain clusters of CpG sequences related to transcription, called CpG islands. These marks are mostly found directly upstream of the start of the gene transcription sequence (Rakyan et al., 2011)*.* Molecular and genetic studies in mammals have shown that DNA cytosine methylation (5 mC) is associated with gene silencing (Jones and Takai, 2001). The methyl residue of methylcytosine is embedded in the major groove of the helix, where many protein regulators meet DNA. Therefore, methylation exerts as its primary mechanism a modulation of contact with transcription factors by attracting or repelling various proteins that bind to DNA (Domcke et al., 2015). A family of methyl-CpG binding domain (MBD) proteins is drawn to and binds to DNA at methylated CpG dinucleotides. MDB proteins induce covalent changes in DNA that have been shown to incorporate repressor protein complexes into promoter regions, contributing to transcriptional silencing (Nan et al., 1998). Furthermore, it is known that CpG methylation regions can also prevent the binding of certain activating transcription factors (Miska and Ferguson-Smith, 2016). Under these concepts, DNA methylation induces transcriptional silencing mediated by repressor proteins or by the absence of activators in the promoter areas with these methylation marks.

Although DNA methylation patterns can be passed from cell to cell, they are not permanent. Changes in DNA methylation patterns can occur throughout life. Some changes may be a physiological response to environmental variations. For example, eating a high-fat diet can change the DNA methylation pattern of some liver enzymes. On the other hand, methylation changes may be associated with pathological processes such as oncogenic transformation or cell aging (Klutstein et al., 2016; Sales et al., 2017).

DNA methylation is catalyzed by the *de novo* DNA methyltransferases (DNMTs), DNMT3A, and DNMT3B. The isoform DNMT1 is responsible for maintaining the methylated residues. DNMT3A plays a relevant role in maintaining cardiomyocyte function (Madsen et al., 2020). The active form of removing methyl groups from DNA can be performed by demethylation involving the action of a family of DNA hydroxylases called methylcytosine dioxygenase (TET) proteins (Bhutani et al., 2011). A passive demethylation process can also change methylation by inhibiting the maintenance of methyltransferase, DNMT1, during cell division. However, DNA methylation patterns fit directly as an epigenetic variable. DNA methylation can also indirectly change gene expression through their close link with other epigenetic mechanisms, such as methylation or acetylation of lysine residues in histone proteins (Coulter et al., 2013).

The clinical relevance of DNA methylation first became apparent after variations in some cancer patients. An increase in the methylation pattern slowed the progression of some tumors in mouse cancer models (Jones and Baylin, 2007). Likewise, low levels of DNA methylation induced by environmental changes were pathologically related to the formation of certain tumors in humans (Van Tongelen et al., 2017). Several other diseases have been linked to gene mutations that encode critical components of the DNA methylation machinery. Therefore, the study of DNA methylation in disease represents an important Frontier in medicine and will contribute to our understanding of the impact of epigenetic modification on life in humans (Schubeler, 2015).

The initial works about DNA methylation events in heart failure were performed on the human myocardium with terminal failure, which reduced DNA methylation of angiogenic genes. Subsequently, it was observed that the patterns of DNA methylation were tightly linked with hypertrophic changes in cardiac cells (Movassagh et al., 2010). A traverse aortic constriction performed in mice closely correlated with different regions of methylation in the genome compared with a control group. Chemical-induced hypertrophy with norepinephrine was associated with global DNA methylation. Further research has demonstrated that the methylation status of cardiac hypertrophy-related genes is modified during cardiac hypertrophy. Transcription factors like GATA4 (GATA binding protein 4) and myocyte enhancer factor 2C (Mef2C) can promote the expression of cardiomyocytes embryonic genes (e.g., ANP, BNP, and β-MHC) (Meder et al., 2017). GATA4, a zinc-finger-containing, DNA-binding transcription factor, is essential for normal cardiac development and homeostasis in humans. Hypermethylation of the GATA4 promoter by persistent nicotine exposure selectively inhibited the expression of GATA4 and induced defects in cardiac cell differentiation (Jiang et al., 2017). The promoters of HEY, a basic helix-loop-helix (bHLH) transcription factor that interacts with HDAC and SR-A (macrophage scavenger receptor 1) promoters, were also hypermethylated in human tissue from different heart failure (Glezeva et al., 2019). A reduction of DNA methylation by a non-nucleoside small molecule that acts as a DNMT inhibitor attenuates the activation of some fetal heart genes induced by transverse aortic constriction (TAC). Treatment with RG108 partially improves contractility, decreases hypertrophy and fibrosis in hearts subjected to TAC (Stenzig et al., 2018).

## Histone modifications

The development of the vertebrate heart requires the covalent modifications of chromatin to induce simultaneous differentiation of several cell types. There are five significant stages of heart development identified in mice, including the cardiac growth stage (E7.75), the linear formation of the heart tube (E8.0), the initiation and construction of the cardiac chambers (E9.5), the maturation and separation of the same (E12.5) to give formation to the valve from E12 until birth finally (Reeves and Whellan, 2010).

Many epigenetic modifications disrupt the basic unit of chromatin, the nucleosome, consisting of 147 base pairs of DNA, wrapped around a histone octamer, which is made up of two copies of each of the four core histones: H2A, H2B, H3, and H4. Unlike those observed with DNA modifications (Kornberg and Lorch, 2020), histone variations are more precise as they depend on the amino acid residues that are modified and the number of added residues. Histone modifications include acetylation, methylation, phosphorylation, ubiquitination, or sumolization (Boeger et al., 2008). The post-transducional (PTM) modifications of histones are achieved by the actions of histone “readers,” “writers” and “erasers.” They are enzymes that either read, add (write) or remove (erase) PTM from the histone proteins. The best-understood regulation of PTM on histones is acetylation, methylation, and phosphorylation (Lizcano and Garcia, 2012). Due to space limitations, we will refer mainly to 2 significant histone PTMs—acetylation and methylation (Rosales et al., 2016; Du et al., 2022). PTMs affect gene expression by inducing a change in chromatin structure that adjusts the profile of DNA binding to transcription factors. Since changes are highly variable, attempts have been made to establish a functional configuration of the effect of variations on histones through a histone functional code. For example, the trimethylation of histone 3 of lysine 9 (H3K9me3) is characteristic of the repression of transcriptional activity and is observed in heterochromatin (Zhao and Garcia, 2015; Willcockson et al., 2021). In contrast, the acetylation of lysine 4 of histone 3 (H3K4Ac) is an activator of gene expression (Du et al., 2022). In the last 30 years, many studies have demonstrated the abilities of proteins with enzymatic capacity to modify the structure of the nucleosome and give way to processes such as DNA replication or transcription. All these enzymatic modifications are reversible; even some configurations, such as the trimethylation of histone 3 in Lysine 9 residues (H3K9me3), considered an irreversible modification, can be modified by (Huang et al., 2014).

The histone acetyltransferases (HAT) induce transcriptional activation because of interference between DNA and histones binding. With the overexpression of CREB-binding protein/p300, a renowned HAT, the mouse developed left ventricular myocyte hypertrophy and cardiac dysfunction (Yanazume et al., 2003). The activation of p300 can induce hypertrophy-responsive through the activation of transcriptional factors like GATA4. Which causes a change in the expression levels of fetal genes, such as ANP, BNP, endothelin-1, and β-MHC, and ultimately leads to cardiomyocyte hypertrophy (Yanazume et al., 2003; Sunagawa et al., 2010). Acetylation of GATA4 made by p300 augments the left ventricle remodeling after myocardium infarction compared to wild-type mice. In contrast, the hearts of the HAT-deficient mutant p300 mice did not exhibit these changes (Miyamoto et al., 2006). The cooperation of p300/GATA4 and acetylation of GATA4 are critical events in cardiomyocyte hypertrophy and the development of heart failure (Takaya et al., 2008). Experimental studies with the inhibition of HAT activity of p300 through Curcumin have shown a significant reduction in models of hypertension and infarct-induced hypertrophy (Sunagawa et al., 2018; Sunagawa et al., 2021).

The histone deacetylation (HDAC) in nucleosomes induces chromatin condensation, which represses transcription by preventing the binding of transcription factors and other components of the transcriptional machinery to a gene promoter and enhancer regions. (Weeks and Avkiran, 2015). HDACs can be classified into three classes based on their homology to yeast histone deacetylase: HDAC I is in the nucleus and include HDAC1, HDAC2, HDAC3, and HDAC8; HDAC II shuttles between the cytoplasm and nucleus and contains two groups HDAC IIa (HDAC4, 5, 7, and 9), mainly located in the nucleus and HDAC IIb (HDAC6, 10) located in the cytoplasm (Gregoretti et al., 2004). HDAC III represents a family that comprises SIRT regulators of transcription, dependent on ATP for their function (Sun et al., 2018).

Class IIa HDCAs inhibit cardiac hypertrophy (Weeks and Avkiran, 2015) by recruiting epigenetic regulators (Bagchi and Weeks, 2019). The nuclear export of class IIa HDACs permits the induction of hypertrophic genes by preventing their repressive interactions with transcription factors and allowing the recruitment of HATs and histone demethylases (Eom et al., 2014; Hohl et al., 2014). Knockout for HDAC IIa members HDAC5 and 9 were sensitive to cardiac stress and developed cardiac hypertrophy in mice with an increase in hypertrophic genes ANP and β-MHC (McKinsey et al., 2002). Nuclear export of HDAC4 can be regulated by by CaMKII (calcium/calmodulin-dependent kinase) phosphorylation, resulting in the activation of MEF2C (myosin enhancer factor 2C), cardiac gene transcription, and induces cardiomyocyte hypertrophy (Zhao et al., 2021).

Concerning class II, class I of HDACs inhibit the cardioprotective and anti-hypertrophic genes. HDAC2 reduction in mice resulted in less sensitivity to hypertrophic stimuli, whereas the mice with Hdac2 overexpression developed cardiac hypertrophy. Models of HDAC class I inhibitors such as Mocetinosta, Valproic acid, Trichostatin A, o Butyrate acid have shown a reduction of cardiac hypertrophy and fibrosis (Bush and McKinsey, 2009; Bradner et al., 2010; Zhao et al., 2012; Kee et al., 2013; Williams et al., 2014). HDAC3 has been involved in developing cardiac hypertrophy in mice. Cardiac-specific inactivation of Hdac3 gene caused cardiac hypertrophy and alteration in fatty acid metabolism (Montgomery et al., 2008). Preclinical studies have demonstrated the efficacy of HDAC1 inhibitors in the therapy of diastolic dysfunction. Ginivostat, an inhibitor of HDAC1 and HDAC2, improves diastolic dysfunction in heart failure models with preserved ejection fraction (Jeong et al., 2018). Additionally, it reduces the proliferation of the extracellular matrix. Givinostat also decreased cardiac fibroblast activation by reducing the presence of BRD4 (bromodomain-containing protein 4), a reader of acetylated histone residues (Travers et al., 2021). These studies were replicated using feline models where it was observed that the pan-HDAC inhibitor suberoylanilide hydroxamic acid (SAHA) improves heart failure with preserved ejection fraction (HFpEF). SAHA decreased left ventricular hypertrophy, diastolic dysfunction, and atrial and pulmonary remodeling (Wallner et al., 2020).

The modifications induced by histone methylases have a different spectrum from those previously observed with the histone acetylases. Because the residues can be mono-di or trimethylated, and the changes can occur in arginine, lysine, or histidine amino acids (Papait et al., 2017). However, the most frequent modifications occur in lysine residues. More than 60 protein lysine methyltransferases (PKMTs) have been characterized in the human genome and classified into those that contain the SET [Su(var)3-9, Enhancer-of-zeste and Trithorax] domain and those that lack this domain (Luo, 2018). Histone methylations can be associated with gene activation or repression, depending on which residue on histone the modification occurred. For example, the H3K4me3 is usually associated with dynamic promoter activity, whereas the tri-methylation of histone 3 lysine 9 (H3K9me3) is linked to the inhibition of transcription (Chen and Dent, 2014).

Methylation of histones also plays a critical role in the progress of HF, as evidenced by the different effects in modulating histone methyltransferase, demethylase, or co-factors in cardiomyopathy mouse models (Papait et al., 2013; Zhao and Garcia, 2015). The euchromatic histone methyltransferase (EHMT2), a histone methylase, is required for cardiomyocyte homeostasis to silence the fetal gene program in the adult heart (Papait et al., 2017). EHMT2 was found to be upregulated during the initial stages of cardiac hypertrophy. Furthermore, chemical inactivation of EHMT2 in transverse aortic constriction mice improved cardiac function and prevented the development of hypertrophy (Papait et al., 2017). PRMT5 (protein arginine methyltransferase 5), which usually is involved in the regulation of cardiac hypertrophy, decreases this cellular process through methylation of arginine in the histone 4 (H4R3me2) (Beacon et al., 2020). PRMT5 deficiency contributes to cell hypertrophy inducing pathological structural cardiac disease and increased SMYD1 (SET and MYND domain Containing) hypertrophy-associated proteins (Tracy et al., 2018; Szulik et al., 2020).

Histone demethylases are a growing group of enzymes that can be classified into two super-families: those belonging to the amine oxidase superfamily, which is dependent on FAD as a co-factor, and those corresponding to the oxygenase superfamily, in which the demethylase activity is dependent on Fe (II) and α-ketoglutarate. This latter superfamily is associated with the presence of a characteristic domain termed Jumonji (Lizcano and Garcia, 2012).

The influence of histone demethylases has two aspects. While LSD1/KDM1A and PHF8/KDM7B have a protective effect on cardiac hypertrophy (Liu et al., 2015; Huo et al., 2021). The rise in KDM4A or KDM3A induces cardiac hypertrophy. Increased function of KDM4A causes an intensification of cardiac hypertrophy in response to pressure overload exposure, mediated by demethylation of histone 3-lysine 9 (H3K9) residues and activation of genes such as ANP, BNP, and four and a half LIM domain (FHL1) which are essential for the development of hypertrophy in rats. An effect was probably performed in conjunction with HDAC4 (Tang et al., 2014; Rosales and Lizcano, 2018).

KDM3A is a demethylase of H3K9me2 residues with a similar effect on the production of cardiac hypertrophy to that detected with KDM4A. However, it was observed that the activity of KDM3A extends over the function of the extracellular matrix-TIMP1 (metallopeptidase inhibitor) and TGFβ (transforming growth factor β). The fact that induces an increase in cardiac fibrosis. A pan-inhibitor of demethylase activity, JIB-04 was shown to have a beneficial effect on hypertrophy and fibrosis triggered by KDM3A (Zhang et al., 2018; Zhang et al., 2022). However, other preclinical studies have shown some different effects (Guo et al., 2022).

At the posttranscriptional level of the three epigenetic regulators, writers and erasers have been reported in cardiac diseases. However, the role of readers has recently been observed in cardiac hypertrophy. Proteins read lysine-acetylated residues and can perform this activity through well-characterized domains called bromodomains (Muller et al., 2011). This domain was the first characterized, and to date, about 40 proteins share the sequences of this domain. One of the best-defined bromodomain families is the BET (Bromodomain and extra-terminal domain) group. BET proteins can bind other proteins with the ability to bind to enhancer regions of gene regulation and trigger a “super” enhancer effect (Borck et al., 2020). There is evidence that BET inhibitors (BETi) exhibit protective effects against pathological cardiac hypertrophy. A pan-BETi, JQ1, ameliorates cardiomyocyte hypertrophy and transverse aortic constriction (TAC)-induced left ventricular hypertrophy in mice (Spiltoir et al., 2013). Another BETi, apabetalone (RVX-208), improves cardiovascular outcomes in patients with diabetes after acute coronary syndrome, suggesting its therapeutic potential for cardiovascular diseases (Ray et al., 2019). Among the four members of the BET family, BRD4 has been widely reported to be involved in the regulation of cardiac hypertrophy. Recently, a BETi (apabetalone) improved the cardiac inflammatory process presented by patients with COVID-19 infection (Mills et al., 2021).

## Non-coding RNA

In gene expression, there is a general concept that in eukaryotes, almost the entire genome is transcribed. However, a low percentage of the RNA transcript can be translated into protein. In the basal state, most of the RNA in human cells consists of non-coding RNA (ncRNA), which includes 80–90% ribosomal RNA (rRNA), 15% transfer RNA (tRNA), and only 7% it is messenger RNA (mRNA), which be able to code for proteins (Cech, 2012). Other ncRNAs correspond to 0.01%; some are miRNAs, siRNAs, or RNAs that regulate the splicing between exons (snRNA) (Mattick and Makunin, 2006). There is an excellent variety in the types of ncRNA that can be present in low concentrations, but their levels can be variable according to the function of the cell.

By general convention, most ncRNAs longer than 200 nucleotides, regardless of whether they have a known function, have been grouped into a category called “long non-coding RNAs” (lncRNAs) (Hombach and Kretz, 2016). These are at levels that are two orders of magnitude lower than mRNA. LncRNAs are involved in regulating protein localization, translation, post-translational modifications, mRNA stability, and chromatin conformation, often through their secondary structure (Zhang et al., 2019).

The miRNA pathway works through the RNA interference machinery (Dicer and Ago proteins). miRNAs typically bind to the 3′ UTR of target mRNAs and repress the mRNAs, thus affecting the protein levels of specific genes (Care et al., 2007). miRNA-133 and miRNA-131 correlate to cardiac hypertrophy (Statello et al., 2021). miRNA-21 plays a role in HF because of the modulation of genes such as MMP2 (matrix metalloprotease-2) and TGFβ receptor III (Gorica et al., 2022). Micro RNAs are very suitable as markers or therapy for some neoplastic diseases. In cardiovascular diseases, global cardiac miRNA profile analysis revealed that miR-217, miR-216a-5p, miR-21-3p, miR-665, and miR-144-3p were upregulated miRNAs in hearts from cardiac heart failure patients (Li et al., 2016). miR-217 is involved in the progression of some cancers, controlling various signaling pathways and anti-oncogenes such as phosphatase and tensin homolog (PTEN) (Hamidi et al., 2022). In cardiac hypertrophy, overexpression of miR-217 induces a functional reduction of the enzymes that regulate histone methylation. In this process, the removal of euchromatic histone methyltransferase (EHMT1/2) decreases the levels of H3K9me2, and increases the expression of fetal genes that codifies for proteins such as ANP, BNP and β-MHC (Thienpont et al., 2017; Nie et al., 2018).

The mitochondrial-derived non-coding RNA predicting cardiac remodeling (*Lipcar*) was associated with left ventricular remodeling. LIPCAR was downregulated early after myocardial infarction but upregulated during later stages. LIPCAR levels identified patients developing cardiac remodeling and were independent of other risk markers associated with future cardiovascular deaths and are in the study as a biomarker of cardiac remodeling and predict future death in patients with heart failure (Kumarswamy et al., 2014; Zhang et al., 2017; Yan et al., 2021). The H19 is an abundant imprinted lncRNA downregulated in HF (Liu et al., 2016). Mechanistically H19 may reduce the expression of proapoptotic genes through the interaction of polycomb repressive complex 2 (PRC2) (Fernandez-Ruiz, 2020). Experimentally H19 gene therapy prevents and reverses pressure-overload-induced HF (Viereck et al., 2020). Cardiac hypertrophy-associated epigenetic regulator (CHAER) is a heart-enriched lncRNA involved in cardiac hypertrophy development by directly binding to the catalytic subunit of PRC2. The PRC2 complex induces methylation of H3K27me3 for the silencing of genes. It was described that the interaction between *Chaer* and PRC2 reduces the methylation of H3K27me in the region of the ANP promoter. Therefore, it may cause an increase in cardiac hypertrophy-related genes (Wang et al., 2016; Wang and Cai, 2017).

LncRNAs can also elicit their function by interfering with the activity of miRNAs. One example is the interaction between lncRNA [cardiac hypertrophy–related factor (CHRF)] and miR-489 (Wang et al., 2014). CHRF was shown to act as a sponge RNA to directly bind to miR-489, an anti-cardiac hypertrophy miRNA. This interaction affects the inhibition of myeloid differentiation primary response gene (myd88) by miR-489 and, therefore, causes cardiac hypertrophy.

## The remodeling of chromatin

Description of chromatin remodelers was initially made in yeast studies where their role as transcriptional regulators was required for sucrose fermentation, among other processes (Neigeborn and Carlson, 1984). They were later described as transcription co-activators that needed energy from ATP to mobilize the nucleosome and allow the agglutination of transcriptional machinery (Pan et al., 2019). The function of these proteins is complex since they enable the nucleosomes to slide, varying their position with the DNA, creating a state of remodeling in which the DNA is more accessible. Still, the histones remain bound and can additionally allow the replacement of histones by histone variants (Gryder et al., 2022).

The first remodeling complexes described in yeast were SWI/SNF, also called BAF (BRG1-associated factors) in mammals. Although there is a variety of proteins that belong to the complexes, a structural homology is conserved in the different species (Clapier et al., 2017). SWI/SNF comprises numerous subunits, including three central or “core” subunits, SMARCB1/BAF47 (actin-dependent transcription regulator, matrix-associated SWI/SNF, subfamily b1), SMARCC1/BAF155, and SMARCC2/BAF170, present in all variants of the complexes. Additionally, two mutually exclusive ATPase subunits, SMRCA4/BRG1 and SMARCA2/BRM (Tolstorukov et al., 2013). Two variants of this complex have been identified that differ based on the subunits composed: the BAF complex contains the subunit ARID1A/BAF250a or ARID1B/BAF250b, and the Polybromo-associated baf complex (PBAF) with the subunits PBRM1/BAF180 and ARID2/BAF200. SWI/SNF is essential during early embryonic development and is also required for the differentiation of many cell types, including myocytes, hepatocytes, and adipocytes (Pedersen et al., 2001; Gresh et al., 2005; Kadoch et al., 2013; Valencia et al., 2019). BAF is usually abundant in the embryonic heart but downregulated in the adult myocardium. Using murine models, it has been observed that a BAF subunit can interact with other epigenetic modulation factors (Han et al., 2014). A cluster of lncRNA named myosin heavy-chain-associated RNA transcript (Mhrt) reduces the increase mediated by the BRG1, an essential ATPase subunit of the BAF complex, which interacts with HDAC and poly ADP-ribose polymerase (PARP) to suppress the α-MHC isoform and activate the fetal β-MHC isoform (Wu and Arora, 2015). In the hypertrophic and failing hearts, the subunits of BAF complexes and their binding partners, HDACs and PARPs, were increased, and the expression of fetal α-MHC has decreased (Hang et al., 2010). Table 1.

**TABLE 1:** Epigenetic mechanisms in the development of hypertrophic cardiomyopathy.

Mechanism	Regulator	Action
**DNA Methylation**	Dnmt1	Gene regulation causes methylation of CpG sites
	Dnmt3a	We are maintaining the cardiomyocyte function
	TET	Genes involved
		ANP, BNP, b-MHC: Embryonic cardiac proteins
		CHF1, MRS1: Involved in the development of hypertrophic obstructive cardiomyopathy
		Dnmt1 inhibitor attenuates cardiac hypertrophy
**Histone Modifications**		
**Writers**		
Histone Acetyltransferases	p300, CBP	Development left ventricular myocyte hypertrophy and cardiac dysfunction
		Increasing acetylation of MEF2 and GATA4
Histone Methyltransferases	EHMT2	EHMT2 was found to be upregulated during the initial stages of cardiac hypertrophy. Increased function of EHMT1/2 protects against pathological cardiac hypertrophy
	PRMT5	Regulation of cardiac hypertrophy decreases this cellular process through methylation of H4R3me2
	PTIP	Misregulation causes cardiac hypertrophy
** Erasers**		
Histone Deacetylases (HDAC)	HDAC Class II	Suppress cardiomyocyte hypertrophy
	HDAC Class I	Inhibition of MEF2 activity (myocyte enhancer factor 2)
		Regulates cardiac metabolism and growth
		In preclinical studies, the inhibition of HDAC class I such as Ginivostat, trichostatin
		A, Valproic Acid, Mocetinosta or SAHA have shown benefits in cardiac hypertrophy and diastolic dysfunction in heart failure with preserved ejection
Histone Demethylases (KDMs)	KDM4A/JMJD2A	Increased function of KDM4A developing cardiac hypertrophy in response to pressure overload exposure, mediated by demethylation of histone 3-lysine 9 (H3K9)
	KDM3A	KDM3A induces cardiac hypertrophy and fibrosis
	KDM7B and KDM1A	Protective effect on cardiac hypertrophy
**Readers**		
Bromo Proteins—BET family	BRD4	Induces Hypertrophic gene expression by binding to the acetylated chromatin, facilitating the phosphorylation of RNA polymerases II and leading to transcription elongation
**Non-coding RNA**	H19	H19 is imprinted LncRNA down regulated in hypertrophy. H19 is a target to reduce cardiac hypertrophy
	Chaer	Chaer, may reduce the expression of proapoptotic genes through the interaction of PRC2
	Mhrt	Mhrt reduce the hypertrophy generated by BRG-HDAC-PARP pathway
	mir-217	miR-217 induces cardiac hypertrophy by reducing EHMT1/2 activity
**Remodeling of Chromatin**	BAF	BAF forms a repressor complex with HDAC and PARP proteins

Dnmt1, DNA, Methyltransferases1; TET, methylcytosine dioxygenase proteins; ANP, atrial natriuretic peptide; BNP, brain natriuretic peptide; β-MHC, Beta-myosin heavy chain; CBP, CREBP-binding protein; CHF1, cardiovascular helix-loop-helix factor 1; MRS1, Macrophage scavenger receptor 1; EHMT2, euchromatic histone methyltransferase; PRMT5, Protein arginine methyltransferase 5; PTIP, Cofactor of H3K4 methylation; HDAC, histone deacetylases; KDM4A, Lysine demethylase 4A; KDM3A, Lysine demethylase 3A; KDM7B, Lysine demethylase 7B; BRD4, Bromodomain-containing protein 4; Chaer, Cardiac hypertrophy-associated epigenetic regulator; PRC2, polycomb repressive complex 2; Mhrt, myosin heavy-chain-associated RNA, transcript; BAF, BRG1-associated factors; PARP, poly(ADP-ribose) polymerase proteins. For further information and references can be found within the text.

## Conclusion

**FIGURE 2:**
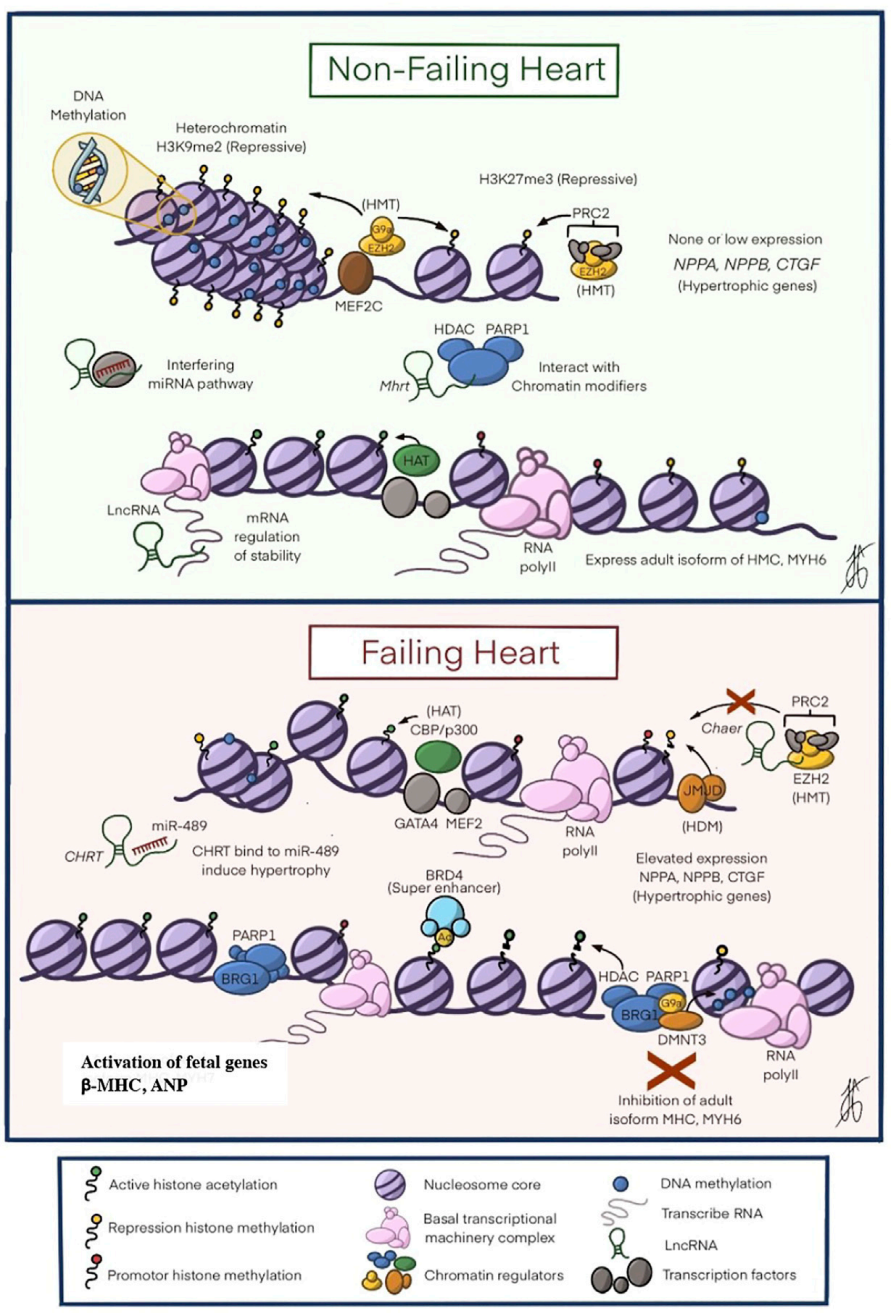
The Epigenetic Mechanisms in Heart Failure. In A shows the perfect balance between the different epigenetic modifications to ensure that the heart develops appropriately. As seen in the figure, there is a greater expression of restrictive modifications: DNA methylation, histone modification (H3K9me2 and H3k27me3), and interaction of non-coding RNA that causes chromatin modifications to modulate down the expression of NPPA, NPPB and CTGF genes related to cardiac hypertrophy. In B shows how the balance above is altered, with a decrease in DNA methylation, an increase in histone acetyltransferases (HAT), PRC2 alterations that prevent histone methylation, as well as the expression of HDACs that lead to the inhibition of essential genes for the cytoskeleton such as MHC and MYH6. All these changes increase the expression of the hypertrophic genes NPPA, NPPB, and CTGF, leading to the development of hypertrophic cardiomyopathy. ATP, adenosine triphosphate; CpG, cytosine-phosphate-guanine; HAT, histone acetyltransferase; HDAC, histone deacetylase; HMT, histone methyltransferase; mRNA messenger RNA; BRD4, Bromodomain-containing 4; β-MHC beta myosin heavy chain beta; PTM post-translational modifications. Modified by Liu, C.-F. et al. J Am Coll Cardiol Basic Trans Science. 2019; 4 (8):976-93.

Cardiac hypertrophy and heart failure are increasing medical challenges due to the increased pathologies committed to their appearance. Currently, there is no specific therapeutic plan for this disease due to the complexity of its etiology. The influence of external factors that induce chromatin modifications has established a language that codes for the expression of genes deciphered in recent years (Figure 2). Modifications that change the function of chromatin readers, writers, or erasers influence the development of cardiac hypertrophy. Advanced and sophisticated tissue engineering approaches allow studying pathological pathways extensively without requiring *in vivo* animal studies, which are more costly, time- and labor-intensive. Epigenomics is becoming a big data science with a potentially enormous effect on our understanding of CVD. Software like the project ENCODE https://www.encodeproject.org/, has growing information about gene expression and epigenetics. Although causality remains established and conflicting evidence exists, novel opportunities may be provided for diagnostic and therapeutic avenues. Epidemiological studies that relate epigenetics to chronic diseases, specifically obesity, have shown alterations in DNA methylation in large population groups. Although these modifications are a consequence of obesity itself rather than its cause. The close relationship of obesity with diabetes mellitus can translate therapeutic measures based on epigenetics. The effect of targeting epigenetic mechanisms appears promising, but these therapies for CVD still await a long way ahead; some difficulties that require special attention are cellular discrimination of epigenetic changes in cardiac tissue, discerning the interaction of the various epigenetic pathways, and determining metabolic regulation in cardiac tissue. A prospective application of the current knowledge base of epigenetic regulation for cardiac diseases is the development of epigenetic biomarkers for diagnosis and prognosis. Creating such comprehensions into epigenetic modifications along the natural history of human cardiac hypertrophy will link strengths for developing personalized medicine.
